# Challenges and opportunities in diagnosing and managing mild traumatic brain injury in rural settings

**DOI:** 10.22605/RRH7241

**Published:** 2022-06-15

**Authors:** Jill Daugherty, Dana Waltzman, Shena Popat, Amy Horn Groenendaal, Maggie Cherney, Alana Knudson

**Affiliations:** 1 Division of Injury Prevention, National Center for Injury Prevention and Control, US Centers for Disease Control and Prevention, Atlanta, GA, USA; 2 Division of Injury Prevention, National Center for Injury Prevention and Control, US Centers for Disease Control and Prevention, Atlanta, GA, USA; 3 NORC at the University of Chicago, Bethesda, MD, USA; 4 NORC at the University of Chicago, Bethesda, MD, USA; 5 NORC at the University of Chicago, Bethesda, MD, USA; 6 NORC at the University of Chicago, Bethesda, MD, USA

**Keywords:** concussion, disparities, injury prevention, traumatic brain injury, USA

## Abstract

**Introduction:**

There is some evidence to suggest that Americans living in rural areas are at increased risk for sustaining a traumatic brain injury (TBI) compared to those living in urban areas. In addition, once a TBI has been sustained, rural residents have worse outcomes, including a higher risk of death. Individuals living in rural areas tend to live farther from hospitals and have less access to TBI specialists. Aside from these factors, little is known what challenges healthcare providers practicing in rural areas face in diagnosing and managing TBI in their patients and what can be done to overcome these challenges.

**Methods:**

Seven focus groups and one individual interview were conducted with a total of 18 healthcare providers who mostly practiced in primary care or emergency department settings in rural areas. Providers were asked about common mechanisms of TBI in patients that they treat, challenges they face in initial and follow-up care, and opportunities for improvement in their practice.

**Results:**

The rural healthcare providers reported that common mechanisms of injury included sports-related injuries for their pediatric and adolescent patients and work-related accidents, motor vehicle crashes, and falls among their adult patients. Most providers felt prepared to diagnose and manage their patients with TBI, but acknowledged a series of challenges they face, including pushback from parents, athletes, and coaches and lack of specialists to whom they could refer. They also noted that patients had their own barriers to overcome for timely and adequate care, including lack of access to transportation, difficulties with cost and insurance, and denial about the seriousness of the injury. Despite these challenges, the focus group participants also outlined benefits to practicing in a rural area and several ways that their practice could improve with support.

**Conclusion:**

Rural healthcare providers may be comfortable diagnosing, treating, and managing their patients who present with a suspected TBI, but they also face many challenges in their practice. In this study it was continually noted that there was lack of resources and a lack of awareness, or recognition of the seriousness of TBI, among the providers’ patient populations. Education about common symptoms and the need for evaluation after an injury is needed. The use of telemedicine, an increasingly common technology, may help close some gaps in access to services. People living in rural areas may be at increased risk for TBI. Healthcare providers who work in these areas face many challenges but have found ways to successfully manage the treatment of this injury in their patients.

## Introduction

A traumatic brain injury (TBI) is caused by a bump, blow, or jolt to the head or a penetrating injury to the head that can affect how the brain works^1^. Lifetime prevalence of TBI, including concussion, is estimated to be 12–30%^2–4^. While all Americans are at risk of experiencing TBIs, there is evidence to suggest that people living in rural areas are at increased risk^5–7^. Research has established that residents of rural areas in the US who survive a TBI are more likely to experience challenges accessing TBI-related services to help in their recovery. This disparity is due to factors such as higher cost for care^8^, less access to Level 1 trauma centers^9^ and specialized TBI care^8,10^, further distance to care^7,8,11^, and not knowing about available services or inability to pay^12^. In fact, a national survey reported that rural Americans live, on average, twice as far from the nearest hospital as Americans living in urban areas^13^. This access barrier makes both receiving initial care for the acute injury as well as follow-up appointments more difficult for rural Americans. Studies also suggest that rural TBI patients may have worse outcomes following TBI than their urban counterparts, such as lower discharge rates, an increase in being functionally dependent, and reporting a lower health status^14,15^.

However, there are also some advantages to living in a rural community. Rural communities are often reported to be tight knit. This connection can allow providers to better know their community members, thereby increasing trust between the patient and provider, and can make it easier to form partnerships with other providers and community-based organizations^16,17^. In particular, personalization of care can be a major advantage to living in a rural community, as rural providers tend to be very familiar with their patients and their families, histories, and circumstances; thus, rural providers are often more able to tailor care to the patient^17^. Cultural differences between urban and rural communities also influence healthcare experiences as rural traditions of self-reliance and community belonging can sometimes facilitate access to care by ‘taking charge’ of one’s condition and diminish vulnerability due to the strength of their community relationships^17^.

The goal of the present study was to gain insight into the day-to-day experience that American healthcare providers who practice in rural areas have in diagnosing, treating, and managing their patients who present with a TBI. A series of focus groups were conducted with these providers to gain information about the challenges they face in their TBI practice, with a goal of informing rural TBI prevention and communication efforts. This study was focused on mild TBI (mTBI), or concussions, historically identified by a Glasgow Coma Scale of 13–15, which make up the majority of TBIs.

## Methods

This qualitative study was carried out from October 2019 to February 2021. The authors designed a semi-structured focus group discussion guide. The information collection was also approved by the Office of Management and Budget for compliance with the requirements of the *Paperwork Reduction Act*.

### Participants

The research team sought a diverse sample of rural providers. The convenience sample was recruited primarily through organization listservs, including the listservs for the National Rural Health Association, National Organization of State Offices of Rural Health, and National Association of State Head Injury Administrators. Providers were selected so that multiple healthcare settings and provider types (physicians (doctor of medicine (MD)/doctor of osteopathic medicine (DO)), nurse practitioners (NPs), physician assistants (PAs), and clinical social workers (CSWs)) were represented in the sample as TBIs can be diagnosed and managed in various settings and by a range of providers. Providers were also sampled so that all four US Census regions were represented. All focus groups were conducted by telephone and scheduled at the providers’ convenience. This approach was chosen as it allowed for flexibility with the providers’ schedules and eliminated any need for travel.

### Procedure

Data were collected by trained moderators (AK, SP, and AH). Each moderator had prior training and experience conducting qualitative research. A semi-structured focus group discussion guide containing 19 semi-structured, open-ended questions was used, with follow-up questions asked when appropriate. The focus group questions were developed based on identified issues in rural care identified in the literature. The full focus group discussion guide (Appendix I) included six main topics: overview of TBI in the rural community; challenges in diagnosing TBI; challenges with treatment and management of TBI; access to care for TBI; challenges with treatment and management of TBI in children, if applicable to a provider’s patient population; and opportunities to enhance TBI diagnosis, treatment, and management. A description of the study purpose was provided in advance; written consent to participate in a 60–90-minute focus group and permission to be audio-recorded were obtained before the start of each focus group. Each focus group included the participants, one moderator, one notetaker, and up to one observer (another member of the research team).

### Data

A total of 17 providers participated in seven focus groups (Table 1); all were composed of two or three participants. An 18th provider participated in a one-on-one interview when they had to reschedule their focus group participation. The majority of the respondents were female, of non-Hispanic White race/ethnicity, and practiced in primary care settings. A total of seven participants practiced in the Midwest, six participants practiced in the South, and five participants practiced in the West. Targeted outreach was conducted through organizations and individuals located in the Northeast; however, no providers identified from the Northeast consented to participate in the study.

### Analysis

A grounded theory approach was employed in which the themes emerged from the data (inductive approach)^18–20^. This approach to content analysis identifies themes that arise directly from the data. The themes are designed to capture the experiences, key challenges, and feedback provided by study participants. The focus groups were recorded and transcribed verbatim. The authors coded and analyzed the transcripts for themes using both NVivo v12 (QSR International; https://www.qsrinternational.com/nvivo-qualitative-data-analysis-software/home) and MaxQDA (VERBI software, 2020; https://www.maxqda.com). The analysis explored emergent themes for the six main topic areas covered in the discussion guide. Codes were compared to verify their descriptive content and to confirm that they were indeed grounded in the data. This article represents a thematic reconstruction of the rural healthcare providers’ responses to the interview questions (Table 2).

### Ethics approval

The NORC Institutional Review Board determined this research to be exempt from full review.

## Results

### Overview of traumatic brain injury in the community

While the self-reported frequency of TBI presentation in the communities in which each provider practiced varied, there were several similarities in the overall burden, common causes of injury, and level of provider preparation for managing TBIs.

#### Mechanism of injury:

The mechanism of injuries (MOI) for the TBIs and concussions seen in these rural providers’ communities were diverse. The most frequently mentioned MOI for the TBIs and concussions were sports. Providers in all eight focus groups cited sports as a common cause of head injury, particularly among their younger patients. Popular contact sports such as hockey, basketball, and football were all specifically named. Two of the providers also noted that they had treated several young people who had concussions sustained during participation in rodeos. The second most commonly mentioned MOI was falls. These injuries were most often seen in the very young and in older adults. The third most common MOI of concussions among these providers’ patients were occupational accidents, such as people who had hit their head while farming, ranching, and working in coal mines. Less commonly, providers spoke of managing TBIs that were due to abuse or assault, motor vehicle crashes, and all-terrain vehicle (ATV) use.

#### How prepared providers feel to diagnose and manage patients with mild traumatic brain injury:

Most of the rural providers who participated in the focus groups felt well prepared to diagnose and manage mTBIs and concussions in their patients. Several providers noted that their comfort and expertise had increased in recent years, due to new state concussion laws, an increased focus in the media, and parents being more aware of the risks of sports- and recreation-related concussions. They also said that training opportunities had increased and improved alongside these developments. For example, one provider noted that, in his state, ‘we have good trauma training in our rural areas – a program that covers it – which is a rural emergency care program and so it’s readily available and large numbers of providers avail themselves in the last 15–20 years to training. I feel that our training helps lead to a more consistent approach.’ Most of the difficulties rural providers face in treating patients with mTBI are, according to the providers, not due to lack of preparation or training but more likely due to the pushback from the patients and their families.

### Challenges in managing traumatic brain injury in rural settings

#### Provider challenges in diagnosing mild traumatic brain injury:

Rural providers stated that lack of awareness among the public is a top challenge in diagnosing mTBI. Providers mentioned that people in the community are often ‘naïve to the signs and symptoms associated with it [concussion]’ and that ‘people are under the impression that you have to always hit your head to get a concussion, which is not the case.’ Another common challenge is pushback or hesitance from athletes, parents, and coaches. As one provider noted:

Athletes, family, and even coaches … have been particularly reluctant to have players see us. There is this perception that once you get diagnosed with an injury you are going to be out of playing and people cannot deal with that.

In addition, the providers reported that certain age groups, such as youth and older adults, present their own unique challenges to diagnose mTBI. For example, they said that in very young children the lack of ability to talk can make diagnosis difficult, while for older adults difficulty in remembering the event, delaying care, and comorbidities make diagnosis challenging.

#### Patient barriers to obtaining initial care following a mild traumatic brain injury:

Focus group participants named several barriers that their patients experienced when deciding whether to obtain care for their suspected mTBIs. First and foremost, the rural providers recognized that transportation was a significant issue for many of their patients. One provider noted, ‘It is not just TBI, transportation is a problem for all medical care in these areas that are spread out.’ Many rural areas have limited options for public transportation and some families do not have personal vehicles they can use to drive to their healthcare providers’ offices. Some patients also do not have the resources to obtain private transportation, such as paying for a taxi. This access barrier is compounded by the long distances to access care.

The second most common barrier to obtaining initial care for their mTBI were patients’ difficulties with cost and insurance. The providers noted that there are sometimes obstacles to getting appropriate diagnostic care for their patients who are uninsured, covered by Medicaid (the USA’s medical expenses assistance program), or those who may have commercial insurance with high out-of-pocket costs (eg deductibles, co-pays, out-of-network charges). One provider spoke about the issue of high-deductible insurance:

High deductible insurances – I think that’s one thing. Sometimes I think particularly in our youth, parents sometimes are reluctant to bring their athlete in if they don’t think it’s that big of a deal, so to speak. If they have a high deductible insurance, they would rather stay home and monitor them so they probably – maybe sometimes wait until their symptoms have gotten bad before they seek health care.

Related, some providers noted that their patients are fearful of having to take time off work to recover, so they choose not to seek care that may result in a potentially serious diagnosis.

Many providers also noted that denial or a lack of recognition of the seriousness of an mTBI was common among their patients. The focus group participants noted that patients may have a preconceived notion of what a TBI is and, if their injury does not fit that definition, they may not think that they have a TBI. For example, several rural providers commented that many of their patients believed that, to sustain a TBI, you have to lose consciousness.

#### Challenges in care management and follow-up:

Providers expressed several difficulties with treating and managing patients in a rural setting. The most common issue was lack of convenient access to care. Rural providers expressed that for complicated or severe cases there was often a limited number of specialists in the community and these specialists typically have to serve a large region. As a result, rural providers at times had to refer patients to larger cities to see a specialist. However, these specialists are often very far away, and this distance represented a barrier to effective treatment. As one provider noted:

I think that is also super-overwhelming for the patient. For us here, we are like, ‘oh go to neurology,’ well the closest one is [in nearest small city] and they’re like ‘I’m not driving [there].’ […]I send a few of the younger kids up to Children’s [hospital]. Their concussion program is phenomenal but that is a four-hour drive for us.

The result is that rural patients often have to take an entire day off from work, which may not be possible and may lead to a lack of follow-up care. Respondents said there can also be long waits for follow-up appointments. As a consequence, management of TBI is sometimes relegated to non-specialists: ‘We don’t have a highly trained specialist, so a lot of this falls onto primary care – like everything does in rural settings.’

Similar to obtaining the initial care for the injury, providers note that cost is a frequent barrier to getting follow-up care. Some patients baulk at the amount of care or the types of specialists that are recommended during their mTBI recovery, due to concerns about being able to pay for it. Even those with insurance have to pay a co-pay or some deductible, which may not be an insignificant cost.

### Opportunities for improvement

#### Benefits of practicing in a rural community:

While there are many challenges to providing TBI care in a rural community, there are also unique benefits. One benefit of rural practice is that because primary care providers tend to treat a broader range of conditions, they have more experience and expertise (compared to less-rural primary care providers) in treating more complicated TBI patients. Other rural providers noted that for some, since they have seen a lot of concussions in their practice, concussions have become a passion and they take time to learn more about how to diagnose, manage, and treat this condition. Additionally, one provider liked the autonomy and the close-knit community commonly found in a rural setting:

In rural areas, it’s all up to the individual doctor and the parents and the child. To me, it’s easier and better there because I can actually see the patient on a daily or once a week or twice a week basis until they’re completely recovered.

#### Traumatic brain injury-related information that would be helpful to improve patient knowledge and awareness:

In general, the focus group participants agreed that their patients are more knowledgeable about TBIs and concussions than they used to be. ‘I think there is a lot more sensitivity about coming in now than back when I started practice.’ Despite this, they noted numerous information gaps and the continued need for public education. The providers spoke often about the need for patients and general public education about what exactly is an mTBI or concussion, what common symptoms are, and the seriousness of this injury. Providers stated that they want their patients to know that mTBI symptoms likely will not disappear in 2 days, and that’s normal. As one provider said:

People have a cavalier attitude about the TBI, not understanding how much long-term negative effect it can have … Education and helping people understand the sequence from a mild TBI to long term problems that can lead to education on that would be helpful in bringing people in and less resistance to being seen.

Providers indicated that they want their patients to be more aware of the risks of re-injury and for care of head injuries to be destigmatized, such as challenging the idea that getting help means you are weak. Additionally, they want short messages that they can convey to patients, and ways to involve families and sports coaches in young athletes’ recoveries.

Some providers thought it would be a good idea to tailor information to student athletes in particular, given that many of the concussions they see are sports-related. They would like to see that youth of pre-high school age and their families are educated about the risks of sports-related concussion. They felt that high school-aged adolescents are receiving adequate education and oversight, but that is less true among younger children. One provider noted that this is important because in some areas of the country children begin contact sports such as hockey at very young ages.

Other providers thought that the focus of public education should be on middle-aged and older adults. Because there has been such a focus in the media and in state laws about sports-related concussions among children, there may be a gap in knowledge about non-sports concussions. These respondents thought the public needed information about other mechanisms of injury, the importance of presenting to a healthcare provider soon after a potential concussion, and the normal course of recovery, which is often longer than patients expect.

#### Clinical training/continuing education that would be helpful:

Some providers had a desire for more clinical education on mTBI, despite generally feeling comfortable diagnosing and managing these injuries currently. One provider noted:

I think one of the things that is common to all of us is we have to have continuing medical education. We all know the basics … the CDC [Centers for Disease Control and Prevention] [could] come out with a program where we could obtain some level of continuing medical education to refresh our knowledge.

Box 1 shows a comprehensive list of topics on which rural providers would like more information and training.

Outside of additional training, several providers mentioned that their TBI practice could benefit from the increased use of telemedicine. This could potentially help to decrease the barriers for those with a suspected concussion to receive care. Two providers also mentioned that the COVID-19 pandemic let them see for the first time how they could integrate telemedicine into their practices:

[Because of COVID-19] we now have access to care through tele[medicine] so that has made an enormous difference as well for us to be able to do neurology appointments. It has been so nice to be able to do that. Concussion appointments through children’s [hospital] – all that kind of stuff – has been able to go online and that has made a huge difference for access to care. Not that I want COVID to still be around but it has been hugely helpful in that respect in being able to get access to care.

And another provider remarked:

We’re really starting to implement the telehealth concept here, so if we could all get on board with that that I feel like [it] could be a very beneficial avenue in these types of situations – but technology, that’s going to take some time before it evolves to that level.

## Discussion

The American healthcare providers who participated in the focus groups were firm in stating that they were comfortable diagnosing, treating, and managing their patients who presented with a suspected TBI. However, they also indicated that they faced many challenges managing TBI in their practice. Across the rural providers in this study, the two main issues identified were a lack of resources and a lack of recognition of the seriousness of the injury among their patient population. As already noted, rural health care can be qualitatively different from urban health care due to limited resources. Resource considerations were a common theme among rural providers and affected multiple areas: lack of transportation (for patients to and from appointments or medical transportation to a local or larger hospital), the need for more concussion training and education for providers and the public, and lack of access to specialists in the community. While these providers felt comfortable and capable in managing TBI in their home practice settings, they also recognized that some of their patients with TBI were at a disadvantage compared to their urban counterparts.

On the patient side, transportation, distance to care, and difficulties with cost of care and insurance were also frequently mentioned as barriers to care. These barriers to care affect many health conditions in rural areas, not just TBI^21^. Graves and colleagues reported that adjusted mean healthcare costs in the 6 months after children experienced a mild TBI were about 10% higher for children living in rural areas than for children living in urban areas^8^. People living in rural areas also have less access to Level 1 trauma centers^9^, have to travel further for healthcare^7,8,11^, and may lack access to transportation that can lead to less healthcare utilization^22,23^. This greater cost of care and more difficulty accessing care is compounded by the fact that rural populations have lower median household incomes and a higher percentage of children living in poverty than urban populations^24^. Additionally, there are higher rates of uninsured individuals residing in rural or non-metropolitan counties^25^.

Focus group participants shared several possible solutions to improve access to TBI specialists, including the use of telehealth. While some rural providers may have been using telehealth in the past to deliver care to patients or to get additional training from specialists, the findings from the present study reveal that the COVID-19 pandemic provided the opportunity for some rural providers to increase their use of telehealth to deliver care to their patients. However, disparities in health care are exacerbated by gaps in access to and availability of technology, such as the internet. According to the most recent Federal Communications Commission broadband report, at the end of 2019 more than 17% of rural residents lack fixed broadband access (25/3 megabits per second (Mbps)), which equates to over 11 million people^26^. While this still represents a large number or rural residents who lack fixed broadband access, the percentage who lack access has dropped by more than 20% since the previous year, which is encouraging. However, access to fixed broadband versus being able to afford fixed broadband are different issues. For example, households with lower income tend to have lower rates of subscription to a fixed broadband service for their home^27,28^. Additionally, incomes tend to be lower in rural areas, and rural counties with lower incomes had a lower subscription rate of home broadband services^27,28^. For mobile broadband, at the end of 2019, less than 10% of rural Americans lacked access to 4G LTE (median speed of 10/3 Mbps)^26^ and approximately 60% of Americans were covered by 5G LTE^29^. Additionally, vulnerable populations including older adults, individuals with disability, and those with limited health literacy or English proficiency, a subset of whom live in remote areas, also experience barriers to telehealth^30–32^. Telehealth has the potential to reduce health disparities and improve health outcomes among rural populations through increased access for communication between patients and clinicians and specialists, and to reduce inconveniences, such as decreased travel time and increased timely follow-up^33–35^. Additionally, telehealth can provide rural providers opportunities to learn from specialists and get further training. Tailoring solutions and mitigating barriers to internet access among rural populations would increase health equity in the use of and access to telehealth.

Many of the other challenges noted by the providers were not unique to the rural setting. The focus group participants remarked that there were still some lingering misconceptions about the injury and a not insignificant amount of denial about the potential seriousness of a TBI. Recent research has found that Americans can correctly identify most common symptoms of concussion and effective ways to prevent concussions^36^. However, as this study shows, there may be a persistent belief that, if one sustains a blow to the head, but does not lose consciousness, then a concussion or TBI did not occur and therefore medical care may be unnecessary. However, the prevalence of losing consciousness after a TBI is low, between 6% and 12%^3,37,38^. This misconception may also contribute to the common failure to recognize the seriousness of the injury.

When asked why those with a suspected concussion did not seek health care, many patients will report that it was because they did not think the injury was serious^39,40^. Even among those who understand the seriousness of a concussion or TBI, there may be an impulse to ignore the injury, or hope it gets better on its own, because of a desire to continue working or playing sports. It is important to be evaluated by a healthcare provider after sustaining a suspected concussion or TBI. A healthcare provider can assess the patient for more severe injury and provide discharge instructions for managing symptoms and returning to activity safely. Even though some of the challenges in treating and managing TBI mentioned by the focus group participants are not unique to the rural areas in which they practice, the consequences may be more acute due to a potentially higher incidence rate and poorer outcomes associated with TBI in rural areas^5–7,14,15^.

There are actions that public health professionals and clinicians can take to educate the public about TBI. Based on the focus group discussions, education is needed for the public at large, and perhaps for parents particularly, about the potential seriousness of concussion and TBI. Specifically, it is important to educate the public about the full range of TBI symptoms, that loss of consciousness is actually quite rare, and that seeking immediate evaluation and care is important. As mentioned, providers practicing in rural areas may benefit from continuing medical education on concussion for patients across the lifespan.

The common mechanisms of injury cited by focus group participants were generally similar to the general population: sports, work-related accidents, and falls^2,41,42^. However, ATV- and rodeo-related TBIs may be unique to rural areas as a mechanism of injury for concussion. ATV use is responsible for over 97 000 emergency department visits annually^43^. Surveys show that ATV users infrequently follow recommended safety behaviors, such as helmet use and not riding with passengers^44^. Less is known about the frequency of rodeo injuries, particularly head injuries. However, similar to ATV usage, helmet use in bull-riding and rodeo events has been shown to be protective against injury and fatality^45^. State and local public health agencies may find partnering with national ATV and rodeo groups to provide concussion education beneficial and impactful.

Developing tailored resources for rural communities may be warranted. For example, developing health communication strategies that include information about multiple ways an individual can sustain a TBI, including sports (eg rodeo), recreation (eg ATVs), and occupational accidents (eg while farming, ranching, and working in coal mines) that are more geared towards those in rural communities, may increase identification of a suspected TBI. Information related to TBI prevention may be topics that would benefit from new resources tailored to and piloted with rural residents to assure that the messages resonate with rural residents.

There were multiple limitations to the present study. The number of respondents was small (*n*=18). Due in part to challenges related to the COVID-19 pandemic, a larger study population was unable to be recruited. With a small sample size, it is likely that additional experiences and viewpoints were not captured. For example, different states have different regulations regarding reporting a suspected TBI and return-to-play. Consequently, practicing in certain states may affect how providers treat their TBI patients. Similarly, some providers had more limited experience with treating and managing TBI in their practice and therefore their responses may not be generalizable to all rural providers. However, because of the repetition of responses that were received, it is believed that the data collected would likely represent the experiences and perceptions of many rural healthcare providers. Future research may wish to replicate this study with a larger number or providers representing more US states to ensure the findings are generalizable to all of the USA.

## Conclusion

Rural residents may be at increased risk for sustaining a TBI and may not seek health care for TBI in their rural communities; however, rural healthcare providers routinely manage TBI treatment for their patients. Effective health communication, tailored for rural communities, about TBI prevention and treatment may be a means to improve the health and wellbeing of all rural residents, especially for those who participate in recreational activities more commonly found in rural settings.

## Figures and Tables

**Table 1:** Focus group participant demographics

Credentials	Census region	Primary specialty	Race/ethnicity	Sex
Medical Doctor	Midwest	Family Medicine	Non-Hispanic White	Male
Physician Assistant – Certified	Midwest	Family Medicine	Non-Hispanic White	Male
Medical Doctor	Midwest	Family Medicine	Non-Hispanic White	Female
Neonatal Resuscitation Program, Care Partner	Midwest	Emergency Medicine	N/A	N/A
Medical Doctor	Midwest	Family Medicine	Non-Hispanic White	Male
Medical Doctor	Midwest	Family Medicine	Non-Hispanic White	Female
Advanced Practice Registered Nurse, Certified Family Nurse Practitioner	Midwest	Family Medicine	Non-Hispanic White	Female
Medical Doctor	South	Family Medicine	N/A	N/A
Bachelor of Science	South	N/A	Non-Hispanic White	Female
Bachelor of Social Work	South	Social Work	Non-Hispanic White	Female
Master of Physician Assistant Studies, Physician Assistant – Certified	South	Emergency Medicine	N/A	N/A
Medical Doctor, Fellow of the American Academy of Family Physicians	South	Family Medicine	Non-Hispanic White	Male
Medical Doctor	South	Family Medicine	N/A	Male
Certified Social Worker	West	Behavioral Health	Non-Hispanic White	Female
Physician Assistant	West	Family Medicine	N/A	N/A
Certified Social Worker	West	Mental Health	Non-Hispanic White	Female
Medical Doctor	West	Family Medicine	Non-Hispanic White	Male
Master of Physician Assistant Studies, Physician Assistant – Certified	West	Family Medicine	Non-Hispanic N/A	N/A

N/A, not available.

**Table 2:** Domains and themes generated from the rural healthcare providers’ focus group interviews and selected quotes

Domain	Themes	Selected quotes
Overview of traumatic brain injury in the community	Many TBIs due to sports, falls, motor vehicle crashes, occupational accidents	*My population has a huge TBI and concussion risk because it’s just the sports, it’s the nature of where we live here, we mountain bike, we ski, we do stupid stuff, so I really had not done a lot of concussion until I moved here and started working here almost four years ago.* *Common causes – it varies somewhat with age. Falls seems to make up the greatest number of mild traumatic brain injury in the older folks and particularly [inaudible] they can deteriorate more rapidly and more severely. Other causes are widely varied, motor vehicles crashes in either car or motorcycle type injuries certainly make up a portion of the younger portion of our people.*
	Providers feel well prepared to treat mild TBIs in patients	*I think the diagnosis and really the treatment is fairly – I feel like our providers have a pretty good grip on that.* *I am fairly comfortable with the mild traumatic brain injury assessment – probably easier than it was years ago because they have imaging, CT scanning imaging readily available 24/7, which years ago we didn’t have and made this whole thing a little bit more difficult. So, the assessment isn’t as difficult in that sense we should feel a little more comfortable.*
	TBI is relatively common injury	*I would say it’s probably a solid handful of times in a given month – to be safe. We do a lot of sporting events and that’s honestly the first thing that pops into my mind- especially the football season. I feel like we are getting those two or three times a week at that point.* *I’m 1–2 per month with falls and injuries, anywhere from pediatric to the old.*
	Increased awareness of TBI in recent years	*I think there has been a shift for us anyway with people recognizing, this is not just traumatic brain injury, but people do interpret injuries as being emergent more often than not, so I agree they tend to show up in the emergency department more. Otherwise in the past they used to show up in the office the next day or the next week. A better-informed public has helped bring people in a little earlier. People seem to view that falls with head injury is something they recognize as potential risk for something worse and they do tend to head towards the emergency department more commonly than they used to.*
Provider challenges in diagnosing TBI	Lack of awareness among public of seriousness of injury	*My barriers right now are delay in presentation due to lack of public awareness, or maybe awareness without the buying into the concussion as being important.*
	Pushback/hesitance from athletes, parents, and coaches	*Athletes, family, and even coaches … have been particularly reluctant to have players see us. There is this perception that once you get diagnosed with an injury you are going to be out of playing and people cannot deal with that.* *I think as far as the diagnosis most of us here would feel comfortable. Our roadblock in this is being able to convince family members, parents especially, and high school athletes of the significance of this and finding a way to get them on board with an absence of participation for some length of time.*
	Young children and older adults more difficult to diagnose	*Children are much more difficult, and the really young, especially falls, motor vehicular accident, etc. because you don’t have the ability to talk, you just go off their history. The physical exam, in mild injury, you can’t tell, children are difficult. They may or may not have any sequela[e] at all, externally, but just that history. And they also have difficulty learning, behaviors, irritability, crying, etc. that really cause major problems later. Those are the issues in the young. The old, of course, they don’t remember, they have difficulty, and they have a higher risk of bleeding four to eight days later. So they’re difficult because of their high risk and understanding the underlying cause.*
Patient barriers to seeking TBI care	Lack of transportation	*It is not just TBI, transportation is a problem for all medical care in these areas that are spread out.*
	Lack of insurance or money	*High deductible insurances – I think that’s one thing. Sometimes I think particularly in our youth, parents sometimes are reluctant to bring their athlete in if they don’t think it’s that big of a deal, so to speak. If they have a high deductible insurance, they would rather stay home and monitor them so they probably – maybe sometimes wait until their symptoms have gotten bad before they seek health care.*
	Adults don’t want to take time off of work	*I would say people that work, I’m thinking of my farmers as much as anything, they just don’t come in when they get a head injury.*
Challenges in managing TBI	Lack of specialists in area	*I think that is also super-overwhelming for the patient. For us here, we are like, ‘oh go to neurology,’ well the closest one is [in nearest small city] and they’re like ‘I’m not driving [there].’ [...]I send a few of the younger kids up to Children’s [hospital]. Their concussion program is phenomenal but that is a four-hour drive for us.*
	Long wait times for follow-up appointments	*I sometimes have patients that have trouble – their primary care unfortunately – well for better or for worse – is an urgent care or a practice where it’s hard to get in. I have run into challenges there where they can’t get in for a week or they say go to the urgent care and the urgent care doesn’t feel comfortable with it and refers them to neuroloqy, which does not happen for several weeks. That would probably be the main problem I see besides insurance.*
	Patients may be unable to afford follow-up care	*I would say framed that way – I would say absolutely finances are going to be a problem. We have patients on high deductible plans. I’m 100% sure that they very much think twice about coming in for everything else so they have to be thinking twice about coming in for a mild traumatic brain injury – rural culture is shake it off, buck up, cowboy kind of thing. I would say the finances are a barrier and that is not just for concussion that is for everything.*
Opportunities for improvement	Benefits of practicing in rural area	*In rural areas, it’s all up to the individual doctor and the parents and the child. To me, it’s easier and better there because I can actually see the patient on a daily or once a week or twice a week basis until they’re completely recovered.*
	Need for public education on TBI	*People have a cavalier attitude about the TBI, not understanding how much long-term negative effect it can have … Education and helping people understand the sequence from a mild TBI to long term problems that can lead to education on that would be helpful in bringing people in and less resistance to being seen.*
	Need for additional clinical training/education	*I think one of the things that is common to all of us is we have to have continuing medical education. We all know the basics – if the CDC [Centers for Disease Control and Prevention] were to come out with a program where we could obtain some level of continuing medical education to refresh our knowledge.*
	Increased use of telehealth/telemedicine	*[Because of COVID-19] we now have access to care through tele[medicine] so that has made an enormous difference as well for us to be able to do neurology appointments. It has been so nice to be able to do that. Concussion appointments through children’s [hospital] – all that kind of stuff – has been able to go online and that has made a huge difference for access to care. Not that I want COVID to still be around but it has been hugely helpful in that respect in being able to get access to care.*

TBI, traumatic brain injury.

## Box 1: Topics on which rural providers would like more information and training.

- Long-term sequelae of concussions
- How to have discussions with coaches, athletic trainers, parents, and children
- How to become stronger advocates for rules of return-to-play for student athletes
- Whether there is standardization of care for TBI and what are proper protocols for different age groups
- Importance of screening for TBI when someone presents for other types of injuries, including orthopedic injuries
- How to delineate mild from moderate TBI, especially those cases that are on the borderline
- How treat older adults with mTBI
- How to manage post-concussion syndrome
- How to successfully re-integrate those with severe TBI back into the community
- How to determine if someone has sustained an mTBI in the more distant past but is causing ongoing symptoms
- Review of helpful TBI screening/daignosis tests such as the vestibuler oculomotor screening assessment (VOMS)
- How to address and treat the more behavioral consequences of TBI, including impulse control issues and difficulties with executive function

## Appendix I: Focus group discussion guide

### A. Overview of Traumatic Brain Injury in the Community

1. Tell us about the risk of concussion or mild TBI in your patient population. How often do you assess a potential concussion or mild TBI in a month?
2. What are the most common causes of mild TBI and concussion among residents in your community?
3. How prepared do you feel to diagnose and manage patients with mild TBI with respect to the training you have received, opportunities for your continuing education, and the frequency with which you are able to treat concussion/mTBI in your patient population?

### B. Challenges in Diagnosis

*Moderator:* First, we are interested in learning about your experience **diagnosing** mild TBI in your patient population.

1. What tools do you use to diagnose mild TBI?
2. What challenges or difficulties do you experience in diagnosing mild TBI? What is the primary challenge you face in diagnosing mild TBI? *Prompts, if needed:*
  - Equipment (e.g., imaging, assessment tools)
  - Staffing/Workforce/Barriers to making referrals
  - Training/Continuing Education
  - Patient perceptions or willingness to seek care (e.g., patients do not want to seek care, do not realize they are hurt, or cannot afford treatment)
  - Other
3. Are any of these challenges or difficulties unique to diagnosis of mild TBI in children and adolescents?
4. How do you manage these challenges?

### C. Challenges in Treatment/Management

*Moderator:* Now that we’ve discussed your experiences with diagnosis, let’s move on to your experiences with treatment and management of mild TBI after a confirmed diagnosis.

1. Are patients diagnosed with mild TBI treated and managed within your practice/organization or are they referred/transferred elsewhere?
  - For patients treated locally: What challenges or difficulties do you face in treating and managing mild TBI? *(eg., training, staffing, systems of care, health insurance coverage, etc).* How are these challenges managed?
  - For patients referred/transferred: We’re going to discuss patient challenges with follow up care in just a minute. The question we have now is what challenges or difficulties do you experience when referring/transferring these patients? *(e.g., coordination with other providers, sharing information, patient’s out-of-pocket costs, health insurance coverage, out of network providers, etc.).* How are these challenges managed?

### D. Access to Care

*Moderator:* We want to learn more about the patient barriers to accessing care that you have observed in your community.

1. What, if any, are the patient barriers to obtaining initial care following a mild TBI? *Prompts, if needed:*
  - Knowledge or understanding of TBI
  - Transportation
  - Cost of care/health insurance status (including out of network providers)
  - Other
2. What, if any, obstacles do patients face during the recovery process for a mild TBI? *Prompts, if needed:*
  - Knowledge or understanding of TBI and the recovery process
  - Knowledge or understanding of TBI and the recovery process among employers, school personnel or coaches
  - Distance to follow-up care
  - Transportation
  - Cost of treatment/health insurance status(including out of network providers)
  - Other

### E. Challenges in Treatment/Management in Children

*Moderator:* Let’s talk specifically about the pediatric population in your community.

1. Some communities have adopted what are called “return to school” or “return to learn” policies or practices that are meant to help children and adolescents successfully transition back to school after experiencing a concussion or mild TBI. Do your local school districts or does your state have any return to school policies or practices following concussions for children?
  - If yes, can you tell us about them?
2. Tell us about the processes you go through with children in regard to returning them to school after they have experienced a mild TBI.
  - Please tell me about interactions have you had with parents or educators about returning children to school after experiencing a mild TBI?
3. Tell us about the processes you go through with children in regard to returning them to playing sports after experiencing a concussion.
  - How do you communicate with parents and coaches about this process?

### F. Opportunities to Enhance TBI Diagnosis and Treatment/Management

*Moderator:* We have discussed the barriers and challenges. We would like to learn more about your thoughts about how to make mild TBI diagnosis, treatment, and management better for rural residents.

1. What mild TBI-related information would be helpful to improve patient knowledge and awareness of TBI in your community?
2. What clinical training/continuing education, if any, would be helpful to improve your mild TBI-related practice?
3. What kind of mild TBI training experiences have you found helpful? These can be informal or formal.
4. What other resources might be helpful to improve your mild TBI-related practice? *Prompts:*
  - Clinical decision support tools
  - Health insurance coverage information
  - Other
5. What do you want the CDC to know about mild TBI in rural communities?
6. Is there any additional information about your experiences with mild TBI and concussions that you would like to share at this time?

Thank you for taking the time out of your schedule to speak with us today. The information you provided, and your experiences, will help inform CDC’s future efforts to decrease disparities related to the diagnosis, treatment, and management of TBI. We appreciate and value your contribution. Thank you.
